# Roof-dependent Atrial Flutter Changed into Single-loop Biatrial Flutter During Ablation

**DOI:** 10.19102/icrm.2022.130306

**Published:** 2022-03-15

**Authors:** Jialin Su, Dominika Zoltowska, Haisam Ismail, Ele Wu, Thomas Wannenburg

**Affiliations:** ^1^Division of Cardiology, University of Florida College of Medicine—Jacksonville, Jacksonville, FL, USA; ^2^Division of Cardiology, Donald and Barbara Zucker School of Medicine at Hofstra/Northwell Health, Hempstead, NY, USA

**Keywords:** Ablation, biatrial flutter, interatrial connection, local activation time mapping, roof-dependent atrial flutter

## Abstract

A single-loop biatrial flutter is an uncommon form of atypical atrial flutter, and it can occur with septal or anterior line ablation in the left atrium (LA). We report a case with a roof-dependent atrial flutter that changed into a single-loop biatrial flutter during roof-line ablation. The activation entered the right atrium (RA) at the septum/fossa ovalis and coronary sinus ostium, exited the RA likely via the Bachmann’s bundle and/or septopulmonary bundle, and entered the LA posterior to the roof line. The biatrial flutter was terminated with linear ablation between the right and left inferior pulmonary veins. RA mapping and biatrial flutter should be considered if roof-dependent atrial flutter slowed down during the roof-line ablation without termination.

## Introduction

Atypical atrial flutter (AFL) mostly occurs in patients with prior cardiac surgery or ablation, especially atrial fibrillation ablation, but can also be secondary to an underlying valvular disease or be *de novo* with or without apparent atriopathy. Atypical AFL uncommonly is biatrial, with the re-entrant loop involving the whole or part of both the right atrium (RA) and the left atrium (LA). Septum ablation for LA flutter is associated with the occurrence of biatrial flutter either during or after the ablation.^1,2^ In this case report, we describe a roof-dependent AFL that changed into a single-loop biatrial flutter during roof-line ablation. It terminated with linear ablation connecting the right inferior vein (RIPV) and left inferior pulmonary vein (LIPV).

## Case summary

The patient is a 60-year-old man who presented with symptomatic AFL. He had a history of typical AFL and atrial fibrillation previously treated with cavotricuspid isthmus (CTI) ablation and pulmonary vein isolation (PVI). He also had a history of coronary artery disease with moderately depressed left ventricular ejection fraction and underwent coronary artery bypass grafting.

**Figure 1:**
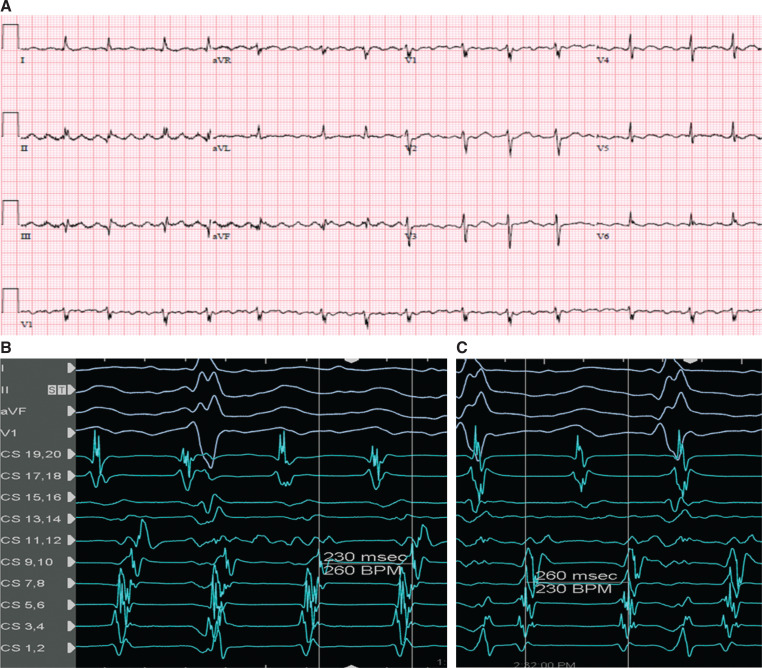
Twelve-lead electrocardiogram of the atypical atrial flutter (AFL) **(A)**, electrogram of AFL with a tachycardia cycle length (TCL) of 230 ms **(B)**, and AFL with a TCL of 260 ms **(C)**. CS 1–10 are in the coronary sinus (CS), and CS 11–20 are in the right atrium around the tricuspid annulus.

**Figure 2:**
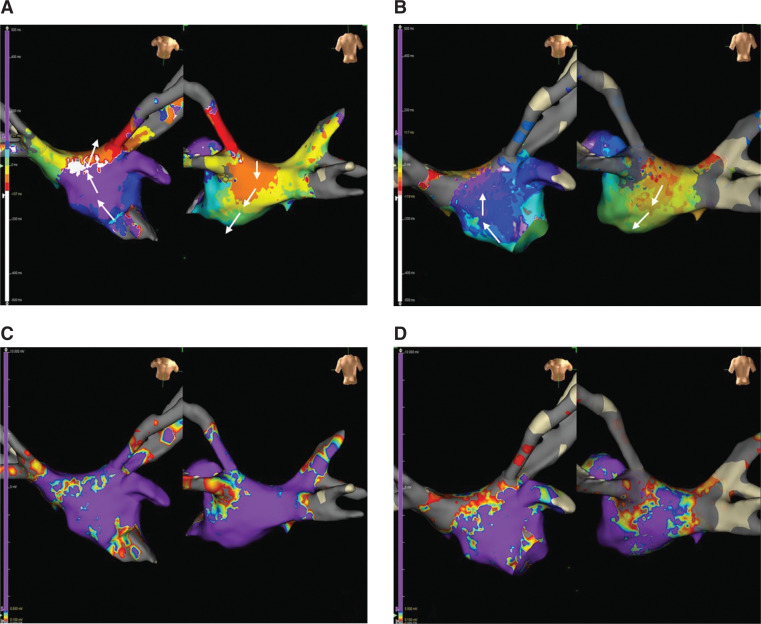
Local activation time and bipolar voltage mapping of the left atrium during atrial flutter with a tachycardia cycle length of 230 ms **(A, C)** or 260 ms **(B, D)**.

**Figure 3:**
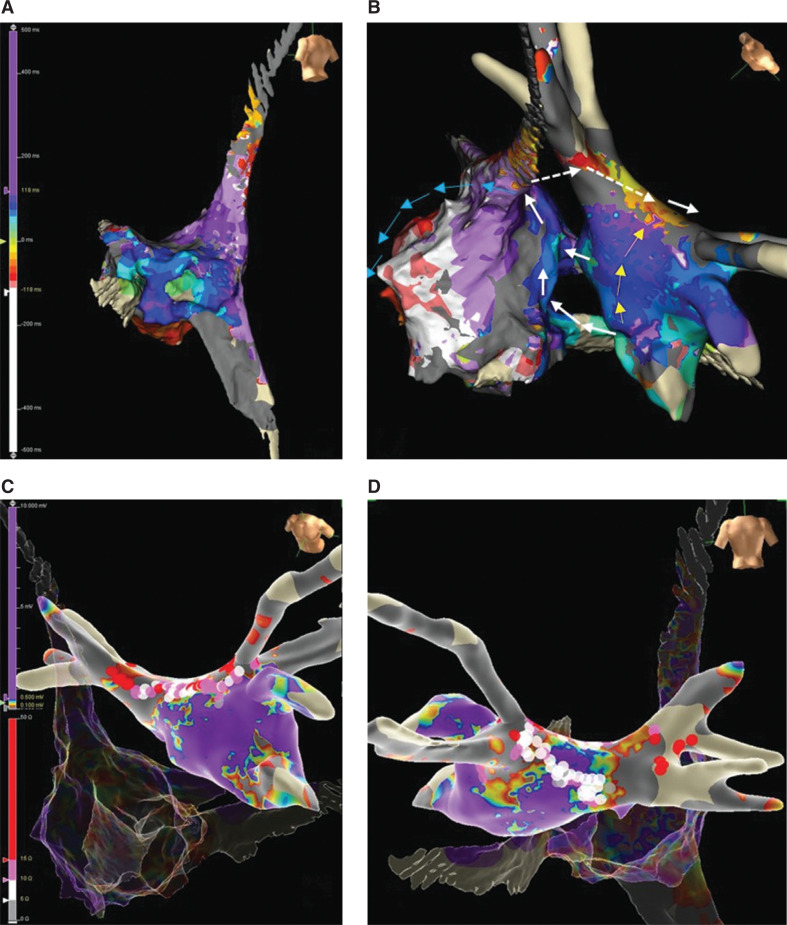
The Local activation time mapping of the right atrium **(A)** or right and left atria combined **(B)** during atrial flutter with a tachycardia cycle length of 260 ms, and the ablation lesion set **(C, D)**.

The patient presented to the electrophysiology laboratory in AFL with a tachycardia cycle length (TCL) of 230 ms. He was not on any anti-arrhythmic medications before the procedure. A duo-decapolar catheter was advanced into the RA and coronary sinus (CS), with the CS 1,2 in the distal CS, CS 9,10 at the proximal ostium CS, and CS 11–12 to CS 19,20 around the tricuspid annulus in the RA. The activation in the CS was earliest at the mid-CS (CS 3,4) **(Figures 1A and 1B)**, and entrainment from the RA showed a post-pacing interval (PPI) of 350 ms with a PPI − TCL of +120 ms, consistent with the LA flutter. A high-density electroanatomic map of the LA and PVs was created (Advisor™ HD Grid Mapping Catheter and EnSite Precision™ 3-dimensional mapping system; Abbott, Chicago, IL, USA) during the AFL. Local activation time (LAT) mapping showed that the activation sequence was from inferior to superior in the anterior LA but from superior to inferior in the posterior LA, and the LAT spans the complete TCL without interruption, consistent with roof-dependent AFL. The bipolar voltage mapping showed normal voltage (>0.5 mV) in the whole LA except the roof, which probably harbored the isthmus of the AFL with conduction velocity deceleration as shown by propagation mapping **(Figures 2A and 2C)**. The diagnosis of roof-dependent AFL was also confirmed by entrainment from both the anterior and posterior LA with a PPI – TCL of less than +20 ms. The right superior PV (RSPV) and left superior PV (LSPV) were connected with entrance conduction into the PV from the LA during AFL. The ablation strategy was to target the isthmus of the AFL by linear ablation from the RSPV to the LSPV and re-isolate both PVs **(Figure 3C)**. During the roof-line ablation, the TCL slowed down to 260 ms with subtle changes in the CS activation **(Figure 1C)**, but did not terminate. The TactiCath^®^ contact force ablation catheter (Abbott) was used for ablation. The ablation parameters were set at 35 W and 30 seconds per lesion, aiming for an impedance drop of >5–10 Ω.

The LA was mapped again during AFL with a TCL of 260 ms. LAT mapping showed the same activation sequence. The roof line was complete without any endocardial gap on the voltage mapping. All 4 PVs were isolated without entrance conduction **(Figures 2B and 2D)**. It was noticed that the LAT of the LA did not span the complete TCL. There was interruption of activation sequence continuity shown by “missing colors” on the LAT map (missed “white” and “red” segments of the TCL). The RA was then mapped. The activation in the right septum was earliest in the mid-septum/fossa ovalis (FO) and CS. The activation at the right mid-septum propagated downward to the CTI, and upward to the SVC, then counterclockwise on the RA free wall and toward the CTI from the lateral RA **(Figure 3A)**. The CTI line from previous ablations was complete, without a gap. The SVC connected with the RA with 1:1 or 2:1 entrance conduction. The combined LAT mapping of the RA and LA showed a single-loop biatrial flutter. The re-entrant circuit was from the mid- to superior right septum, then back to the LA posterior roof, likely via the Bachmann’s bundle (BB) and/or the septopulmonary bundle (SPB), from superior to inferior in the posterior LA, from inferior to superior in the anterior LA, and entering the RA via the mid-septum and CS (indicated by white arrows in **Figure 3B**). Entrainment from the superior right septum showed that the PPI − TCL was +20 ms, indicating that the right superior septum was in the re-entrant loop. Entrainment at the inferior right septum showed that the PPI − TCL was +80 ms, indicating that the inferior right septum was a bystander. The LA septum and anterior LA were also bystanders of the activation loop (indicated by yellow arrows in **Figure 3B**). The activation in the RA free wall was from superior to inferior as a remote bystander (indicated by blue arrows in **Figure 3B**). The LA posterior and inferior walls were in the inner loop of this biatrial flutter. Linear ablation from the RIPV to the LIPV was performed with gradual prolongation of the TCL and termination of the AFL **(Figure 3D)**. The patient was discharged the next day in sinus rhythm and without anti-arrhythmic medication. He was followed up at 1 and 3 months without recurrent atrial arrhythmias.

## Discussion

Atypical AFL mostly occurs with prior cardiac surgery or ablation, and this case had a history of both cardiac surgery (2 cardiac bypass surgeries) and ablations (CTI and PVI ablations). The AFL with a TCL of 230 ms was a single-chamber (LA), single-loop re-entrant loop across the roof. The isthmus of the AFL was at the roof, considering that there was a low-potential region and propagation deceleration across the roof. The roof line targeting the isthmus did not terminate the AFL but changed to a slower biatrial flutter with a TCL of 260 ms.

Single-loop biatrial flutter is an uncommon form of atypical AFL. It was reported to be 0.5%–2.1% in patients with atypical AFL.^1,2^ There are 3 types of biatrial flutter based on the re-entrant circuit. Type 1 is both peri-mitral and peri-tricuspid, type 2 is peri-mitral with the right septum in the re-entrant circuit, and type 3 utilizes the right and left septum.^2^ All of them have electrical obstacles located in the septum as the critical component supporting the biatrial flutter. Anterior line ablation is associated with biatrial flutter due to its disruption of the electrical conduction on the left septum. Biatrial flutter can occur during the ablation procedure of both clockwise and counterclockwise peri-mitral flutter, which can change into a single-loop biatrial flutter without termination, or can be induced after the termination of the peri-mitral flutter.^3^ Sometimes, biatrial flutter occurs in patients with a past medical history of LA septum ablation.^2,4^

This is the first case report we know of where a roof-dependent AFL could also change into a biatrial flutter after roof-line ablation. The entrance of activation from the LA into the RA was via the lower septum/FO and CS, while the exit from the RA was from the right superior cavoatrial junction. The activation entered the dome of the LA posterior to the roof line, likely via the BB and/or SPB, which had insertion in the LA posterior to the roof line and thus bypassed the roof-line ablation. BB is the most robust epicardial interatrial connection across the anterior interatrial groove, branches across the LA dome and toward the LA appendage. SPB is composed of subepicardial fibers arising from the interatrial groove beneath the BB and fanning out to pass the dome and the posterior wall of the LA.^4–6^ We also noticed that there was still some “missing color” (red and white) even with biatrial mapping, indicating that there was interruption of activation sequence continuity and a small portion of the re-entrant circuit of the biatrial flutter was not endocardial. The electrical connection via BB could be epicardial. Lu et al. reported a case with an LA roof–dependent AFL, which continued after intact roof and floor line endocardial ablation without gaps. Epicardial mapping showed that there was epicardial conduction via BB, and the roof-dependent AFL terminated with epicardial BB ablation.^7^ Epicardial mapping during AFL with a TCL of 260 ms for this case probably will confirm the epicardial conduction and add the “missed colors” to the LAT mapping.

Electrical connection in between the RA and LA normally is very robust, with endocardial, subendocardial, subepicardial, and epicardial insertion in a wide and complex area. The electrical connection includes BB, SPB, CS, and anterosuperior and posteroinferior connection around the FO on the septum. This is based on both anatomy and mapping in the human heart during sinus rhythm or atrial pacing.^4,6,8^ Theoretically, if there is an electrical obstacle on the left septum or anterior LA proximal to the most distal insertion of the RA to LA electrical connection via the BB or SPB, either due to prior surgical incision or prior or current ablation including anterior or septal LA ablation as the most commonly reported, biatrial flutter can possibly occur. This is supported by this case, with the BB and/or SPB insertion sites in the LA being posterior to the roof line. There are more different types of single-loop biatrial flutter if different entrance/exit sites in the RA are addressed.

High-density mapping with both LAT and bipolar voltage mapping combined with targeted entrainment are all important to delineate the re-entrant circuit of the AFL and to identify which atrium or which part of the RA or LA is actively involved in the re-entrant circuit (including an inner loop, an outer loop, and an isthmus) and which is passively activated and being a bystander. In this case, the superior right septum was in the re-entrant circuit. The inferior RA septum and RA free wall were passively activated and were bystanders of the macro–re-entrant loop.

Characterization of different components, the propagation, and the re-entrance would be very helpful in designing an ablation lesion set. Either the entrance/exit sites in the RA or LA,^8–10^ or the isthmus of the AFL if it is identified, can be targeted. Targeting the entrance site might be easier than targeting the exit site, considering that the activation of the entrance site into an atrium tends to be less diffuse and the propagation is similar compared to focal activation with centrifugal propagation. The entrance in the RA in this case was at the mid-septum/FO and CS. The FO was not a good target considering the concern of causing a left to right shunt by ablation. The entrance in the LA was posterior to the roof line with a likely component of epicardial BB conduction, and endocardial ablation may not be transmural and would not terminate the AFL. The posterior and inferior walls of the LA were both in the biatrial re-entrant circuit. There was no significant deceleration or abnormal electrogram along the re-entrant loop, but the distance between the isolated LIPV and RIPV was short. We did ablation from the RIPV to LIPV and the biatrial flutter terminated.

The isthmus of the biatrial flutter can possibly be in the RA or LA. In Kitamura et al.’s study, 7 of 9 biatrial flutters were terminated by ablating on the isthmus, which were identified in either the RA or LA.^2^ Ablation in the CS or vein of Marshall for LA peri-mitral flutter,^10,11^ endocardial ablation of SPB,^12^ and epicardial ablation of BB for roof-dependent LA flutter that had failed endocardial ablation^7^ were reported. These strategies are potential options for biatrial flutter ablation after high-resolution LAT and voltage mapping supplemented by targeted entrainment.

## Conclusion

Roof-dependent AFL can change into biatrial flutter during the ablation if the ablation line is proximal to the distal insertion of the interatrial connection bundle in the LA. High-resolution LAT and bipolar voltage mapping combined with targeted entrainment are critical in identifying the re-entrant circuit, the entrance into and exit from the RA. RA mapping should be considered if roof-dependent AFL changes into a different AFL with a longer TCL during ablation, especially if the LAT range in the LA is much shorter than the TCL and/or with interruption of activation sequence continuity. Ablation at the entrance or exit site in either the RA or LA or ablation of the potential isthmus of the re-entrant circuit are options to terminate the biatrial flutter.
